# De-Ritis Ratio Improves Long-Term Risk Prediction after Acute Myocardial Infarction

**DOI:** 10.3390/jcm7120474

**Published:** 2018-11-23

**Authors:** Matthias Steininger, Max-Paul Winter, Thomas Reiberger, Lorenz Koller, Feras El-Hamid, Stefan Forster, Sebastian Schnaubelt, Christian Hengstenberg, Klaus Distelmaier, Georg Goliasch, Johann Wojta, Aurel Toma, Alexander Niessner, Patrick Sulzgruber

**Affiliations:** 1 Division of Cardiology, Department of Internal Medicine II, Medical University of Vienna, 1090 Vienna, Austria; Matthias.steininger@meduniwien.ac.at (M.S.); max-paul.winter@meduniwien.ac.at (M.-P.W.); lorenz.koller@meduniwien.ac.at (L.K.); feras@el-hamid.de (F.E.-H.); forsterstefan@gmx.at (S.F.); christian.hengstenberg@meduniwien.ac.at (C.H.); Klaus.distelmaier@meduniwien.ac.at (K.D.); georg.goliasch@meduniwien.ac.at (G.G.); johann.wojta@meduniwien.ac.at (J.W.); aurel.toma@meduniwien.ac.at (A.T.); Patrick.Sulzgruber@meduniwien.ac.at (P.S.); 2 Division of Gastroenterology and Hepatology, Department of Internal Medicine III, Medical University of Vienna, 1090 Vienna, Austria; thomas.reiberger@meduniwien.ac.at; 3 Vienna Hepatic Hemodynamic Laboratory, Medical University of Vienna, 1090 Vienna, Austria; 4 Department of Emergency Medicine, Medical University of Vienna, 1090 Vienna, Austria; sebastian.schnaubelt@meduniwien.ac.at

**Keywords:** acute coronary syndrome, De-Ritis ratio, AST, ALT, long-term prognosis

## Abstract

Background: Recent evidence suggested levels of aspartate aminotransferase (AST), alanine transaminase (ALT), and AST/ALT ratio (De-Ritis ratio) were associated with a worse outcome after acute myocardial infarction (AMI). However, their value for predicting long-term prognosis remained unknown. Therefore, we investigated the prognostic potential of transaminases on patient outcome after AMI from a long-term perspective. Methods: Data of a large AMI registry including 1355 consecutive patients were analyzed. The Cox regression hazard analysis was used to assess the impact of transaminases and the De-Ritis ratio on long-term mortality. Results: The median De-Ritis ratio for the entire study population was 1.5 (interquartile range [IQR]: 1.0–2.6). After a median follow-up time of 8.6 years, we found that AST (crude hazard ratio (HR) of 1.19 per 1-SD [95% confidence interval (CI): 1.09–1.32; *p* < 0.001]) and De-Ritis ratio (crude HR of 1.31 per 1-SD [95% CI: 1.18–1.44; *p* < 0.001]), but not ALT (*p* = 0.827), were significantly associated with long-term mortality after AMI. After adjustment for confounders independently, the De-Ritis ratio remained a strong and independent predictor for long-term mortality in the multivariate model with an adjusted HR of 1.23 per 1-SD (95% CI: 1.07–1.42; *p* = 0.004). Moreover, the De-Ritis ratio added prognostic value beyond N-terminal pro-B-Type Natriuretic Peptide, Troponin T, and Creatine Kinase. Conclusion: The De-Ritis ratio is a strong and independent predictor for long-term mortality after AMI. As a readily available biomarker in clinical routine, it might be used to identify patients at risk for fatal cardiovascular events and help to optimize secondary prevention strategies after AMI.

## 1. Introduction

Acute Myocardial Infarction (AMI) remains the leading cause of mortality in patients with coronary artery disease (CAD) and the most prominent cause of death in both developing and developed countries [1,2]. Therefore, an individualized risk assessment for potentially fatal major cardiac adverse events (MACE) constitutes a crucial diagnostic approach in order to ensure a reduction of readmission rates and overall mortality in this high-risk patient population [3]. The concept of biomarkers for both diagnosis and prognosis gains importance in the current era of personalized medicine. This rapidly extending field calls for the availability of easily assessable prognostic markers in order to conveniently identify patients at risk. 

In this regard, transaminases such as aspartate aminotransferase (AST) and alanine transaminase (ALT) are easily assessable values in clinical practice and, most importantly, routinely available [4]. Several investigations highlighted liver dysfunction as a common accompaniment in patients with cardiovascular disease, even examining the predictive value of transaminases on morbidity and mortality in a cardiovascular patient population [4,5,6]. However, since most of those studies are referring to patients with chronic heart failure, only a few were conducted on individuals with evident CAD or AMI. Based on recent evidence, transaminases such as AST and ALT may represent predictive markers for patient outcome after an acute cardiac event [4,5,6,7,8,9]. Considering that ALT predominantly mirrors liver-specific dysfunction and AST proved to be elevated after ischemic cell death of several other tissues, including kidney, skeletal muscle, or even brain, the AST/ALT (De-Ritis) ratio might mirror a surrogate marker for ischemic end-organ damage in the acute phase of AMI. Since the De-Ritis ratio might therefore add discriminatory power on the risk assessment of patients after AMI, no attention has been paid to its prognostic effect, leaving a major gap of evidence. Filling this gap of knowledge, we aimed to investigate the prognostic potential of the De-Ritis ratio on the patient outcome after AMI from a long-term perspective. 

## 2. Experimental Section

### 2.1. Methods

#### 2.1.1. Study Population

A detailed study protocol has already been described elsewhere [10]. In short, patients presenting with AMI admitted between December 1996 and December 2009 to the Vienna General Hospital—a university-affiliated tertiary center with a high-volume 24-hour cardiac catheter laboratory—were included in this clinical registry. AMI was defined as an ST-elevation myocardial infarction (STEMI) or a non-ST elevation myocardial infarction (NSTEMI) in accordance to the 2015 guidelines of the European Society of Cardiology (ESC) [11,12]. The presence of heart failure was defined as the presence of heart failure related symptoms (NYHA class ≥II) or reduced left-ventricular ejection fraction <50%. The study protocol complies with the declaration of Helsinki and was approved by the local ethics committee of the Medical University of Vienna (EK 159/2011).

#### 2.1.2. Data Acquisition and Follow-Up

Patient characteristics were assessed via the patients’ electronic medical records of the Medical University of Vienna by specially trained chart reviewers at the time of hospital admission. Data were inserted into a predefined data record abstraction form for further analysis of the registry. 

Blood samples were taken at the time of hospital admission, immediately prior to coronary angiography, and processed in accordance to local laboratory standards (Department of Laboratory Medicine, Medical University of Vienna, Vienna, Austria). Routine laboratory sampling included measurement of serum AST (normality range: Male 17–59 U/L female 14–36 U/L) and ALT (normality range: Male 21–72 U/L, female 9-52 U/L) activity by enzymatic kinetic assays (Cobas C System—Roche Diagnostics, Basel, Switzerland). The De-Ritis ratio was calculated as the ratio of AST and ALT [12].

#### 2.1.3. Outcome Measures

Patients were followed prospectively until the primary study endpoint was reached. Cardiovascular mortality was chosen as the primary study endpoint. The patients’ cause and date of death were collected by screening the national registry of death until January 2017 via the Austrian Registry of Death (Statistics Austria, Vienna, Austria). Postmortem examinations were available in 38% of deceased patients. The cause of death was defined by the physician that determined death for every deceased individual within the present analysis according to the International Statistical Classification of Disease and Related Health Problems 10th Revision. Additionally, the respective cause of death was validated by postmortem examinations, which were available in 38% of deceased patients. Causes of death were defined according to the International Statistical Classification of Disease and Related Health Problems 10th Revision. We chose that the ICD-10 codes as follow for our definition of cardiovascular death: I08, I10, I11, I13, I20, I21, I22, I23, I24, I25, I34, I42, I47, I48, I49, I50, I51, and I63.

### 2.2. Statistical Analysis

Continuous data are shown as median and interquartile range (IQR) and compared using the Kruskal–Wallis test. Categorical parameters are presented as counts and percentages and analyzed using the Mantel–Haenszel-χ^2^ test. The Cox regression hazard analysis was used to assess the influence of AST, ALT, and the De-Ritis ratio on cardiovascular mortality. Results were presented as a hazard ratio (HR) and the respective 95% confidence interval (CI). HRs of continuous variables refer to an increase per one standard deviation (1-SD). Variables were log-transformed prior to their inclusion in the model. In the multivariate regression analysis, HRs were adjusted for potential confounders due to their association with mortality as follows: Age, male gender, body-mass index, hypertension, hypercholesterolemia, diabetes mellitus type II, smoking, STEMI, acute revascularization, family history in coronary vessel disease (CVD), liver function failure, renal function failure, heart failure, chronic obstructive pulmonary disease (COPD), previous AMI, maximum Troponin-T values, C-reactive protein values, Butyrylcholinesterase values, gamma-GT values, estimated glomerular filtration rate (eGFR) at admission, and cardiogenic shock. Cumulative survival in De-Ritis tertiles was examined by Kaplan–Meier curves (log-rank test). A classification and regression tree (CART) analysis was applied to determine optimal cut-off values for the De-Ritis ratio in order to classify groups at high and low risk for cardiovascular mortality. Analysis regarding the detection of a novel prognostic biomarker was conducted in accordance to recent recommendations [13]. The discriminatory power of AST, ALT, and the De-Ritis ratio was evaluated using Harrell’s C-Statistic. The category-free net reclassification improvement (NRI) and integrated discrimination increment (IDI) were calculated to estimate an improvement in individual risk prediction for the addition of the De-Ritis ratio to established risk factors. Considering the purpose of the present analysis, the discrimination and calibration of the presented data has been validated by NRI and IDI, which is recommended for the detection of prognostic biomarkers and illustrated by Cook and co-workers [14].

A sample size of more than 1300 individuals, including an overall probability of an event of 40%, allows detecting a risk factor with an HR of 1.2 (power 80%; alpha 0.05). Statistical significance was defined by two-sided *p*-values < 0.05. Statistical analyses were performed using SPSS 24.0 (IBM SPSS, Armonk, New York, NY, USA) and STATA 11.0 (STATA-Corp, College-Station, Texas, TX, USA). 

The datasets gathered and analyzed during the current study are available from the corresponding author on reasonable request.

## 3. Results

### 3.1. Distribution of De-Ritis Ratio and Baseline Characteristics

Detailed baseline characteristics of the entire study population and stratified by tertiles of the De-Ritis ratio are shown in Table 1 and Table 2. In short, the median age of the entire study population was 63 years (IQR: 43–81), including 796 (58.7%) male participants. The median AST and ALT levels were 42 U/L (26–97) and 19 U/L (29–49), with a resulting median De-Ritis ratio of 1.5 (1.0–2.6). 

We observed a strong direct association of the De-Ritis ratio with age (*p* < 0.001), female gender (*p* < 0.001), and STEMI (*p* < 0.001). Additionally, the De-Ritis ratio showed a strong correlation with both maximum Troponin-T values (*r* = 0.262; *p* <0.001) and maximum creatine kinase values (*r* = 0.264; *p* < 0.001). Interestingly, while the De-Ritis ratio was inversely associated with the estimated glomerular filtration rate (eGFR; *r* = −0.245; *p* < 0.001), the strongest correlation was found with NT-proBNP (*r* = 0.358; *p* < 0.001) and C-reactive protein (*r* = 0.166; *p* < 0.001).

### 3.2. De-Ritis Ratio and Risk Prediction—Discrimination and Reclassification

After a median follow-up time of 8.7 years, corresponding to 11,046 patient years, a total of 554 (40.9%) individuals died, including 414 (30.5%) deaths of cardiovascular causes. We found that AST (crude HR of 1.19 per 1-SD [95% CI: 1.09–1.32; *p* < 0.001]) and the De-Ritis ratio (crude HR of 1.31 per 1-SD [95% CI: 1.18–1.44; *p* < 0.001]) were significantly associated with cardiovascular mortality, while we observed no effect of ALT (crude HR per 1-SD of 0.98 [95% CI: 0.89–1.10; *p* = 0.827]). However, while the significant association of AST was lost (adj. HR of 1.04 per 1-SD [95% CI: 0.72–1.02; *p* = 0.608]) after adjustment for confounders in the multivariate model, De-Ritis remained a strong and independent predictor for long-term cardiovascular mortality, with an adjusted HR of 1.23 per 1-SD (95% CI: 1.07–1.42; *p* = 0.004) (see Table 3).

Similar results were observed for all-cause mortality—both AST and the De-Ritis ratio were significantly associated with mortality. While, the predictive potential of AST was lost within the multivariate model, the De-Ritis ratio still proved a significant association. However, the predictive value of the De-Ritis ratio was comparably lower for all-cause mortality than for cardiovascular mortality (Table 3).

Of note, the predictive potential of the De-Ritis ratio remained stable and irrespective of the date of study enrollment during the extensive observation period (12/1996-06/2004: HR of 1.55 (95% CI: 1.26–1.91) *p* < 0.001; 06/2004-06/2007: HR of 1.23 (95% CI: 1.10–1.52) *p* = 0.036; 06/2007-12/2009: HR of 1.71 (95% CI: 1.29–2.25) *p* < 0.001).

**Figure 1:**
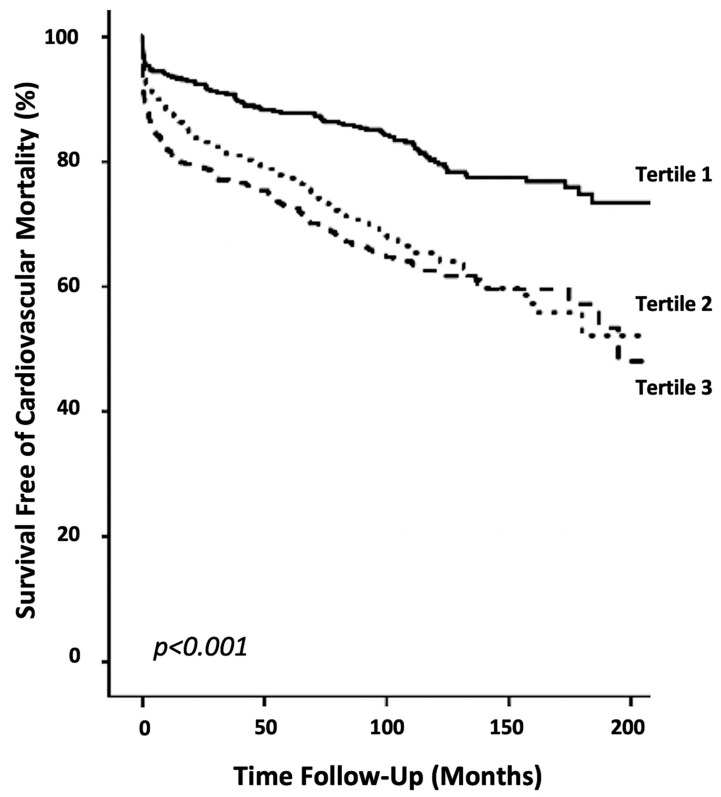
Survival curves of cardiovascular mortality. Kaplan–Meier plots showing cardiovascular mortality within tertiles of the De-Ritis ratio (*p* < 0.001).

The Kaplan–Meier survival curves for tertiles of the De-Ritis ratio were plotted and compared using a log-rank test (*p* < 0.001; Figure 1). In addition to the De-Ritis ratio, only age and NT-proBNP remained independently associated with cardiovascular mortality in the multivariate model. A classification and regression tree (CART) analysis determined a De-Ritis cut-off value of ≤1.2 as low risk for cardiovascular mortality and >1.2 as high risk. The C-statistic for the De-Ritis ratio was 0.61, with 0.53 and 0.46 for AST and ALT, respectively. Since NT-proBNP represents a routinely available and powerful prognostic marker for outcome in acute coronary syndrome and its measurement augments the prognostic information of clinical risk scores, it was chosen as a marker for comparison. NT-proBNP alone presented with a C-statistic of 0.62 and to predict cardiovascular mortality. A lower discriminatory potential was observed for Troponin-T (C-Statistic: 0.52) and for Creatinine Kinase (C-Statistic: 0.54). The combination of NT-proBNP and the De-Ritis ratio significantly increased the discriminatory power to 0.65. Those results were confirmed by improvements in the net reclassification index (NRI; 23.4%; *p* = 0.019) and integrated discrimination improvement (IDI; 0.13; *p* = 0.022). Similar findings were observed for the addition of the De-Ritis ratio to Troponin-T (C-Statistic: 0.59; NRI: 15.9% [*p* = 0.009]; IDI: 0.54 [*p* = 0.01]) and Creatine Kinase (C-Statistic: 0.60; NRI: 19.9% [*p* = 0.001]; IDI: 0.65 [*p* = 0.005]).

## 4. Discussion

To the best of our knowledge, the present study represents the largest one with the longest follow-up that investigated the impact of transaminases on patient outcome after AMI. Since we observed that the independent effect on mortality of both AST and ALT was lost during the follow-up, the De-Ritis ratio remained a strong and independent prognosticator for cardiovascular mortality from a long-term perspective. Since values for the De-Ritis ratio are routinely available in clinical practice, they can easily be used as biomarkers for risk stratification in order to ensure a personalized treatment approach and secondary prevention. The respective improvement of long-term risk stratification seems crucial for this highly vulnerable patient population in order to reduce hospital readmission rates and fatal cardiovascular events. Therefore, the illustrated predictive value of the De-Ritis ratio might help in identifying patients at risk. A routine evaluation of the De-Ritis ratio during the hospitalization of an acute event may improve secondary prevention by closer monitoring, regular follow-up visits, and possibly aggressive pharmacotherapeutic management at the time of discharge. Since values for the De-Ritis ratio are routinely available in clinical practice, they can easily be used as biomarkers for risk stratification in order to ensure a personalized treatment approach and secondary prevention. 

### 4.1. The Impact of Transaminases on Patient Outcome after AMI

Recent data indicated that transaminases might mirror a suitable risk-marker for outcome after acute cardiac events. In this regard, Gao and co-workers found that both AST and ALT were associated with early all-cause mortality in patients presenting with STEMI undergoing PCI. Moreover, they observed that outcomes were significantly worse when both AST and ALT levels were in the ≥95th percentile [4]. Similarly, Moon et al. illustrated in a small cohort study of 456 participants that AST independently predicted all-cause mortality in a more sensitive manner than other established cardiac risk-markers. Of note, they were able to illustrate a strong association of both elevated AST and ALT values and reduced left ventricular ejection fraction after the acute event [13]. In line, Lofthus and co-workers found that both AST and ALT were independent predictors for poor clinical outcomes and all-cause mortality within 30 days [14]. Several other analyses investigated independently the isolated effect of ALT on 90-day mortality after cardiogenic shock [15]. 

Since all available studies in literature elucidated the effect of transaminases exclusively in STEMI patients, we extended the current knowledge by showing that the predictive potential of the De-Ritis ratio was a suitable risk predictor in an unselected general AMI patient population, showing no influencing by type of AMI (interaction term: *p* = 0.756).

Moreover, as already mentioned above, all previous investigations targeted short-term mortality as their primary endpoint, including the maximum follow-up time of 3.5 years. Of note, while the predictive potential of AST and ALT on mortality has been confirmed in several studies, no attention has been paid to the De-Ritis ratio, leaving a major gap of knowledge.

Filling this lack of evidence, we were able to illustrate that the De-Ritis ratio has a strong and independent effect on outcome, even from an extensive long-term perspective, showing follow-up periods of up to 20 years in several individuals. Interestingly, the observed predictive potential of both AST and ALT in recent studies was lost during our long-term follow-up, indicating that their prognostic value in CAD may only be limited to a short-term perspective. Considering the results of recent investigations and the findings of the present analysis, especially the De-Ritis ratio might be a suitable predictor for mortality after AMI, mirroring a potential end-organ damage. Of note, a preexisting hepatocellular damage or myopathies might have a major impact on the individual De-Ritis ratios. However, since the used multivariate model was adjusted for liver function failure (including hepatopathies), the presented predictive potential of the De-Ritis ratio is free of any confounding by a preexisting hepatocellular damage.

### 4.2. De-Ritis Ratio as a Potential Marker for End Organ Damage in the Acute Phase

The De-Ritis ratio was initially described in 1957 and subsequently confirmed to be a useful indicator of the underlying liver disease etiology [16,17]. Lower De-Ritis ratios suggest viral hepatitis (ratio <1; ALT predominance) and higher ratios suggest structural liver cell damage, such as alcoholic liver disease (ratio >1; AST predominance). However, due to the relatively short half-life of AST (18 h) compared to ALT (36 h), one has to carefully assess the time course and aggressiveness of disease for a correct interpretation of the De-Ritis ratio [18]. Interestingly, nonalcoholic fatty liver disease (NAFLD), a liver disease that has been shown to impact on cardiovascular morbidity, is also associated with an elevated AST/ALT ratio that, in turn, reflects liver fibrosis and predicts long-term complications [19,20]. 

It is well established that organ perfusion of individuals presenting with AMI is impaired, depending on the extent of myocardial damage and its impact on cardiac contractility. Acute myocardial ischemia has a strong impact on the left ventricular function, which results in low cardiac output and also contributes to systemic hypoperfusion [6]. Considering the findings of previous studies on individuals with chronic heart failure, the findings of elevated liver enzymes were ascribed to chronic hypoxia-induced liver injury. However, the manifestation of liver damage usually occurs only later after AMI, so this might not explain an isolated AST increase with concomitant low—more liver-specific—ALT values. Therefore, a different approach needs to be postulated, which explains the elevated De-Ritis ratio values in the acute phase of AMI. 

AST is a mitochondrial enzyme and is known to occur not only in the liver, but also in the skeletal muscle, myocardial and kidney tissue, as well as the brain. In contrast, ALT is predominantly expressed by hepatocytes and therefore represents a more specific marker for hepatocellular injury. Considering those pathophysiological mechanisms, an isolated increase of AST and, therefore, leading to an elevated De-Ritis ratio suggests (at least an additional) nonhepatic source. Considering those findings, the De-Ritis ratio therefore potentially mirrors end-organ damage during the acute phase of AMI and the associated systemic hypoperfusion [21,22,23]. 

The impact of butyrylcholinesterase (BChE) and cardiac mortality after AMI has been the subject of recent research. BChE is used in basic laboratory examination as an indicator for liver function and their ability for protein-synthesis [9,15,22,23]. Goliasch and Sulzgruber et al. demonstrated a strong inverse association between BChE activity and mortality [9,24]. According to those findings, we were able to demonstrate an inverse association of BChE values and De-Ritis that potentially constitutes the product of impaired hepatic perfusion. Since BChE represents a sensitive value for hepatic dysfunction, it might mirror ischemic hepatopathy already at an early stage of the AMI. The parallel inverse association with bilirubin—as a value for liver synthesis function—fosters this assumption.

Additionally, the De-Ritis ratio proved to be associated with impaired renal function via strong inverse correlation with estimated glomerular filtration rate (eGFR). Of note, the De-Ritis ratio was also significantly associated with cardiogenic shock, markers for cardiac strain (NT-proBNP), and inflammation (CRP). Those associations support our assumption that the De-Ritis ratio mirrors a surrogate marker for end-organ damage via impaired systemic perfusion during AMI. During the past few decades, the understanding of the pathogenesis of ischemic heart disease extended and certain markers for both local and systemic inflammation proved to have a pivotal role in development and disease progression of atherosclerosis [25,26,27]. Immunological proceedings and inflammation are known as a major pathophysiological pathway in cardiovascular disease. Similarly, elevated levels of pro-inflammatory cytokines at baseline are closely associated with the occurrence of a fatal cardiac event in both the general population as well in coronary heart disease. Therefore, systemic inflammation can be both the cause and the consequence of cardiovascular disease [28,29]. In this regard, the De-Ritis ratio has been proposed to mirror a post-ischemic inflammatory response during the acute phase of AMI. The “ischemic hepatitis” as an extensive form of hepatocellular damage during AMI represents a well-established and highly clinically relevant consequence of cardiac dysfunction that proved to have a causative role of long-term liver damage, and also cardiovascular morbidity [30]. Especially metabolic liver diseases, such as nonalcoholic steatohepatitis (NASH), can be a driver for cardiac mortality. This represents a new epidemic of liver diseases associated with the increasing prevalence of the metabolic syndrome [31]. Most importantly, those mentioned stressors within ischemic hepatitis, NASH and metabolic syndrome, proved to have one thing in common: They are all closely linked to both systemic and local inflammatory conditions that might be crucially involved in cardiovascular disease progression after AMI, remodeling, and fibrosis of cardiac tissue. In hepatic disease—mostly indicated by elevated levels of aminotransferases—danger or death-associated molecular patterns (DAMPs) are released. DAMPS are decompartmentalized cellular structures that may be passively released by necrotic hepatocytes or actively by viable or apoptotic parenchymal cells [32]. DAMPs comprise endogenous, ectopic proteins, nucleic acids, adenosine triphosphate (ATP), or mitochondrial compounds. Sterile hepatic and systemic inflammation might be fueled by these DAMPs. Interestingly, and maybe relevant to the long-term prognostic value of the De-Ritis ratio, a second wave of DAMPs might trigger subsequent or secondary inflammatory responses triggered by the activation of Kupffer cells (KCs), the attraction and activation of monocytes and neutrophils [33,34]. Taken together, these mechanisms might all promote systemic sterile inflammation that may not only impact cardiovascular function and/or recovery, but also other end-organ damage in patients after myocardial infarction. In this regard, it is well known that the use of statins exerts a relevant impact on prognosis in patients with cardiovascular disease through several biological processes—mainly based on their anti-inflammatory potential [35,36,37]. However, we found that the median De-Ritis ratio value for patients presenting with statin therapy (1.42 [0.87–2.46]) was comparable to statin naïve patients (1.42 [0.96–2.59]; *p* = 0.387) Similarly, a statin therapy at admission did not modify the predictive potential of the De-Ritis ratio (*p* for interaction: 0.705).

### 4.3. A Novel Biomarker for Cardiac Tissue Damage?

A more hypothesis-generating approach is built on the fact that AST is—in contrast to ALT—also evident in cardiac tissue and was found to be released after ischemic myocardial tissue damage [38,39]. In the first half of the 20th century, AST and ALT have already been in the spotlight of clinical cardiology, when both parameters were used as diagnostic markers to identify patients with AMI. However, after the introduction of high sensitivity cardiac markers, such as troponins and creatine kinase (CK), transaminases lost their clinical application in this purpose. As evident in the literature, CK correlates closely to the actual extent of myocardial damage of the myocardial ischemia in the acute event [2,25,38]. Interestingly, we observed a strong correlation of the De-Ritis ratio with maximum CK values. Those findings are in line with previous investigations and build the basis for the hypothesis that the De-Ritis ratio might represent a marker for the extent of myocardial tissue damage in AMI. However, this issue needs to be addressed in detail in future investigations.

### 4.4. Limitations

The major limitation of the current analysis represents its single center setting. However, having a large number of participants enrolled and through the extensive follow-up, we achieved a high primary endpoint rate and an extensive cumulative observation period of up to 11,499 patient-years, which ensured sufficient statistical power. Unfortunately, we were not able to present data on the patients Killip class, extent of revascularization (culprit vs. complete), and time until revascularization.

## 5. Conclusions

The De-Ritis ratio proved to be a strong and independent predictor for mortality after AMI from a long-term perspective. The De-Ritis ratio revealed prognostic superiority over AST and ALT even after comprehensive adjustment for potential confounders and added additional prognostic value to available and well-established cardiovascular risk markers. Since there is an overwhelming increase of biomarker studies in the field of especially cardiovascular disease with varying quality and questionable clinical unitality, the evaluation of novel biomarkers needs to follow stringent rules and regulations. Considering the recommendations of Ahmad et al. for the evaluation of novel prognostic biomarkers, including accurate methodological and statistical procedures and comparison to state-of-the-art biomarkers, our results fulfill the mentioned quality criteria [26]. 

Especially in the era of personalized medicine, the assessment of easily assessable biomarkers is of utmost importance to identify patients at risk for MACE after AMI. As a routinely available value in clinical practice, the De-Ritis ratio can easily be used for risk stratification in order to ensure a personalized treatment approach and aggravated secondary prevention. 

## Figures and Tables

**Table 1:** Baseline characteristics.

		1st Tertile (*n* = 452)	2nd Tertile (*n* = 452)	3rd Tertile (*n* = 451)	*p*	*r*	* p
De–Ritis Ratio, ratio (IQR)		0.8 (0.6–1.0)	1.5 (1.3–1.7)	3.6 (2.6–5.0)	**<0.001**		
AST, U/L (IQR)		29 (17–42)	35 (26–57)	107 (65–215)	**<0.001**		
ALT, U/L (IQR)		36 (22–60)	24 (17–38)	29 (18–50)	**<0.001**		
Age, years (IQR)		64 (40–72)	71 (44–83)	75 (45–83)	**<0.001**	0.291	**<0.001**
Gender (male), *n* (%)		326 (72.1)	252 (55.8)	218 (48.3)	**<0.001**		
Body mass index, kg/m^2^ (IQR)		27.6 (25.1–30.4)	25.8 (23.8–28.9)	25.3 (23.3–28.1)	**<0.001**	–0.212	**<0.001**
Systolic Blood Pressure, mmHg (IQR)		127 (112–140)	127 (113–145)	124 (110–140)	**0.031**	–0.056	**0.047**
Diastolic Blood Pressure, mmHg (IQR)		76 (68–84)	75 (63–81)	71 (62–80)	**0.001**	–0.096	**0.001**
Heart rate, bpm (IQR)		75 (65–86)	76 (66–89)	78 (66–90)	0.159	0.061	**0.031**
Cardiogenic Shock, *n* (%)		54 (11.9)	45 (9.9)	40 (8.8)	0.125		
Previous AMI, *n* (%)		88 (19.5)	99 (21.9)	72 (15.9)	0.176		
Vessel Disease					**0.001**		
	1–VD, *n* (%)	201 (40.8)	151 (30.6)	141 (28.6)			
	2–VD, *n* (%)	101 (34.1)	94 (31.8)	101 (34.1)			
	3–VD, *n* (%)	109 (28.7)	134 (35.3)	137 (36.1)			
STEMI, *n* (%)		181 (40.0)	242 (53.5)	261 (57.9)	**<0.001**		
Stenting, *n* (%)		321 (71.0)	302 (66.8)	308 (68.1)	0.351		
Fibrinolysis, *n* (%)		77 (17.0)	60 (13.3)	54 (11.9)	0.074		
Hypertension, *n* (%)		300 (66.4)	303 (67.0)	308 (68.3)	0.571		
Diabetes mellitus, *n* (%)		96 (21.2)	91 (20.1)	94 (20.8)	0.870		
Hypercholesterolemia, *n* (%)		322 (71.2)	277 (61.3)	255 (56.5)	**<0.001**		
Renal function failure, *n* (%)		29 (6.4)	29 (6.4)	48 (10.6)	**0.019**		
Chronic heart failure, *n* (%)		21 (4.6)	29 (6.4)	29 (6.4)	0.256		
Current smoker, *n* (%)		285 63.1)	212 (46.9)	212 (46.9)	**<0.001**		
Family history of CVD, *n* (%)		177 (39.2)	138 (30.6)	148 (32.8)	**0.042**		
Peak–Troponin T, µg/L (IQR)		1.3 (0.3–4.1)	1.4 (0.5–3.9)	3.1 (1.3–6.2)	**<0.001**	0.262	**<0.001**
Peak–CK, U/L (IQR)		448 (161–1393)	553 (218–1327)	1010 (508–2166)	**<0.001**	0.264	**<0.001**
Peak–LDH, U/L (IQR)		338 (235–611)	368 (278–600)	551 (381–811)	**<0.001**	0.302	**<0.001**
Quick Test % (IQR)		96 (80–108)	90 (80–101)	90 (75–104)	**0.001**	−0.107	**<0.001**
Fibrinogen mg/dL (IQR)		374 (324–442)	391 (335–470)	418 (349–490)	**<0.001**	0.133	**<0.001**
Gamma–GT U/L (IQR)		38 (23–61)	32 (19–54)	26 (17–44)	**<0.001**	–0.183	**<0.001**
Butyrylcholinesterase, U/L (IQR)		7.0 (5.6–8.6)	6.8 (5.5–8.2)	6.5 (5.4–7.9)	**0.005**	–0.086	**0.002**
Total Bilirubin, mg/dL (IQR)		0.49 (0.36–0.75)	0.55 (0.38–0.82)	0.64 (0.46–0.89)	**<0.001**	0.173	**<0.001**
eGFR, (IQR)		101.9 (67.6–122.0)	65.5 (45.5–104.2)	66.7 (45.2–103.2)	**<0.001**	–0.245	**<0.001**
C–reactive protein, mg/dL (IQR)		0.5 (0.4–1.2	0.5 (0.4–1.3)	1.0 (0.5–2.8)	**<0.001**	**0.166**	**<0.001**
Creatinin, (IQR)		1.05 (0.93–1.21)	1.07 (0.92–1.31)	1.03 (0.85–1.30)	**0.045**	–0.020	0.451
NT–proBNP, (IQR)		519 (163–3548)	986 (281–5033)	2230 (1036–6543)	**<0.001**	0.475	**<0.001**
LVEF <40% at discharge, *n* (%)		37 (8.2)	61 (13.5)	80 (17.7)	**<0.001**		

Categorical data are presented as counts and percentages and analyzed using a test for linear association (Maentel–Haenszel-chi-square-test). Continuous data are presented as median (interquartile range) and analyzed using the Kruskal–Wallis test. The association of continuous variables with De-Ritis ratio was assessed using the Spearman–Rho correlation coefficient. AST = aspartate aminotransferase, ALT = alanine transaminase, AMI = acute myocardial infarction, STEMI = ST-elevation myocardial infarction, CVD = coronary vessel disease, LVEF = Left ventricular ejection fraction, eGFR = estimated Glomerular Filtration Rate.

**Table 2:** Median De–Ritis values and its impact on mortality within categorical variables.

	Yes	No	*p-*Value	Crude HR (95% CI)	*p-*Value
Gender (male)	1.3 (0.8–2.2)	1.7 (1.1–3.0)	**<0.001**	1.32 (1.17–1.44)	**<0.001**
STEMI	1.7 (1.1–2.9)	1.3 (0.8–2.5)	**<0.001**	1.22 (1.07–1.51)	**0.002**
Stenting	1.4 (0.9–2.7)	1.6 (1.0–2.5)	0.285	1.39 (1.22–1.38)	**<0.001**
Thrombolysis	1.3 (0.8–2.4)	1.5 (1.0–2.6)	**0.005**	1.26 (0.99–1.56)	0.060
Previous AMI	1.4 (1.0–2.2)	1.5 (1.0–2.8)	0.159	1.35 (1.13–1.63)	**0.001**
Cardiogenic shock	2.3 (1.3–4.7)	1.0 (0.6–2.1)	**<0.001**	1.47 (1.11–1.63)	**0.007**
Hypertension	1.5 (0.9–2.6)	1.5 (1.0–2.7)	0.897	1.29 (1.16–1.95)	**<0.001**
Diabetes mellitus	1.5 (1.0–2.6)	1.5 (1.0–2.5)	0.937	1.24 (1.05–1.44)	**0.011**
Hypercholesterolemia	1.4 (0.9–2.5)	1.7 (1.1–2.9)	**<0.001**	1.33 (1.19–1.47)	**<0.001**
Renal function failure	1.9 (1.1–4.4)	1.4 (0.9–2.5)	**<0.001**	1.02 (0.80–1.49)	0.853
Chronic heart failure	1.6 (1.1–2.7)	1.4 (1.0–2.7)	**<0.001**	1.49 (1.12–1.99)	**0.006**
Family history of CVD	1.4 (0.9–2.5)	1.5 (1.0–2.8)	0.052	1.32 (1.09–1.99)	**0.003**
LVEF <40% at discharge	1.8 (1.2–3.2)	1.4 (0.9–2.5)	**<0.001**	1.07 (0.87–1.59)	0.497

De–Ritis ratio values are presented as median and IQR. P–values for comparison of De–Ritis ratio values within categorical variables using Mann–Whitney–U test. Cox proportional hazard model for the association of De–Ritis ratio on long–term mortality within subgroups. Hazard ratios (HR) for continuous variables refer to a 1–SD increase.

**Table 3:** Unadjusted and adjusted effects on cardiovascular mortality.

	Crude HR (95% CI)	*p*-value	Adjusted HR (95% CI) *	*p-*Value
**AST**	1.21 (1.10–1.32)	**<0.001**	1.15 (1.00–1.33)	0.051
**ALT**	0.99 (0.89–1.09)	0.987	0.98 (0.85–1.33)	0.811
**De-Ritis Ratio**	1.31 (1.19–1.44)	**<0.001**	1.24 (1.08–1.44)	**0.002**

Cox proportional hazard model. Hazard ratios (HR) for continuous variables refer to a 1-SD increase. * The multivariate model was adjusted for: Age, male gender, body-mass index, hypertension, hypercholesterolemia, diabetes mellitus type II, positive smoking status, STEMI, acute revascularization, family history in CVD, liver function failure, renal function failure, heart failure, COPD, previous AMI, maximum Troponin-T values, C-reactive protein values, Butyrylcholinesterase values, gamma-GT values, eGFR at admission, and cardiogenic shock at admission.

## References

[1] Timmis A., Townsend N., Gale C., Grobbee R., Maniadakis N., Flather M., Wilkins E., Wright L., Vos R., Atlas Writing Group (2018). European society of cardiology: Cardiovascular disease statistics 2017. Eur. Heart J..

[2] Roger V.L. (2007). Epidemiology of myocardial infarction. Med. Clin. North Am..

[3] McAlister F.A., Lawson F.M., Teo K.K., Armstrong P.W. (2001). Randomised trials of secondary prevention programmes in coronary heart disease: Systematic review. BMJ.

[4] Gao M., Cheng Y., Zheng Y., Zhang W., Wang L., Qin L. (2017). Association of serum transaminases with short- and long-term outcomes in patients with st-elevation myocardial infarction undergoing primary percutaneous coronary intervention. BMC Cardiovasc. Disord..

[5] Batin P., Wickens M., McEntegart D., Fullwood L., Cowley A.J. (1995). The importance of abnormalities of liver function tests in predicting mortality in chronic heart failure. Eur. Heart J..

[6] Allen L.A., Felker G.M., Pocock S., McMurray J.J., Pfeffer M.A., Swedberg K., Wang D., Yusuf S., Michelson E.L., Granger C.B. (2009). Liver function abnormalities and outcome in patients with chronic heart failure: Data from the candesartan in heart failure: Assessment of reduction in mortality and morbidity (charm) program. Eur. J. Heart Fail..

[7] Poelzl G., Ess M., Mussner-Seeber C., Pachinger O., Frick M., Ulmer H. (2012). Liver dysfunction in chronic heart failure: Prevalence, characteristics and prognostic significance. Eur. J. Clin. Invest..

[8] Alvarez A.M., Mukherjee D. (2011). Liver abnormalities in cardiac diseases and heart failure. Int. J. Angiol..

[9] Sulzgruber P., Koller L., Reiberger T., El-Hamid F., Forster S., Rothgerber D.J., Goliasch G., Wojta J., Niessner A. (2015). Butyrylcholinesterase predicts cardiac mortality in young patients with acute coronary syndrome. PLoS One.

[10] Sulzgruber P., El-Hamid F., Koller L., Forster S., Goliasch G., Wojta J., Niessner A. (2017). Long-term outcome and risk prediction in patients suffering acute myocardial infarction complicated by post-infarction cardiac rupture. Int. J. Cardiol..

[11] Van de Werf F., Bax J., Betriu A., Blomstrom-Lundqvist C., Crea F., Falk V., Filippatos G., Fox K., Huber H., Kastrati A. (2008). Management of acute myocardial infarction in patients presenting with persistent ST-segment elevation: The task force on the management of ST-segment elevation acute myocardial infarction of the european society of cardiology. Eur. Heart J..

[12] Roffi M., Patrono C., Collet J.P., Mueller C., Valgimigli M., Andreotti F., Bax J.J., Borger M.A., Brotons C., Chew D.P. (2016). 2015 ESC guidelines for the management of acute coronary syndromes in patients presenting without persistent st-segment elevation: Task force for the management of acute coronary syndromes in patients presenting without persistent st-segment elevation of the european society of cardiology (ESC). Eur. Heart J..

[13] Moon J., Kang W., Oh P.C., Seo S.Y., Lee K., Han S.H., Ahn T., Shin E. (2014). Serum transaminase determined in the emergency room predicts outcomes in patients with acute ST-segment elevation myocardial infarction who undergo primary percutaneous coronary intervention. Int. J. Cardiol..

[14] Lofthus D.M., Stevens S.R., Armstrong P.W., Granger C.B., Mahaffey K.W. (2012). Pattern of liver enzyme elevations in acute ST-elevation myocardial infarction. Coron. Artery Dis..

[15] Jantti T., Tarvasmaki T., Harjola V.P., Parissis J., Pulkki K., Sionis A., Silva-Cardoso J., Kober L., Banaszewski M., Spinar J. (2017). Frequency and prognostic significance of abnormal liver function tests in patients with cardiogenic shock. Am. J. Cardiol..

[16] De Ritis F., Coltorti M., Giusti G. (1957). An enzymic test for the diagnosis of viral hepatitis; the transaminase serum activities. Clin. Chim. Acta.

[17] Wroblewski F. (1958). The clinical significance of alterations in transaminase activities of serum and other body fluids. Adv. Clin. Chem..

[18] Kamimoto Y., Horiuchi S., Tanase S., Morino Y. (1985). Plasma clearance of intravenously injected aspartate aminotransferase isozymes: Evidence for preferential uptake by sinusoidal liver cells. Hepatology.

[19] Sorbi D., Boynton J., Lindor K.D. (1999). The ratio of aspartate aminotransferase to alanine aminotransferase: Potential value in differentiating nonalcoholic steatohepatitis from alcoholic liver disease. Am. J. Gastroenterol..

[20] Neuschwander-Tetri B.A., Clark J.M., Bass N.M., van Natta M.L., Unalp-Arida A., Tonascia J., Zein C.O., Brunt E.M., Kleiner D.E., McCullough A.J. (2010). Clinical, laboratory and histological associations in adults with nonalcoholic fatty liver disease. Hepatology.

[21] Botros M., Sikaris K.A. (2013). The de ritis ratio: The test of time. Clin. Biochem. Rev..

[22] Hall P., Cash J. (2012). What is the real function of the liver ‘function’ tests?. Ulster Med. J..

[23] Giannini E.G., Testa R., Savarino V. (2005). Liver enzyme alteration: A guide for clinicians. CMAJ.

[24] Goliasch G., Haschemi A., Marculescu R., Endler G., Maurer G., Wagner O., Huber K., Mannhalter C., Niessner A. (2012). Butyrylcholinesterase activity predicts long-term survival in patients with coronary artery disease. Clin. Chem..

[25] Pohl J., Hendgen-Cotta U.B., Stock P., Luedike P., Baba H.A., Kamler M., Rassaf T. (2017). Myocardial expression of macrophage migration inhibitory factor in patients with heart failure. J. Clin. Med..

[26] Lopez-Candales A., Hernandez Burgos P.M., Hernandez-Suarez D.F., Harris D. (2017). Linking chronic inflammation with cardiovascular disease: From normal aging to the metabolic syndrome. J. Nat. Sci..

[27] Libby P., Ridker P.M., Hansson G.K. (2009). Inflammation in atherosclerosis: From pathophysiology to practice. J. Am. Coll. Cardiol..

[28] Fuhrmann V., Kneidinger N., Herkner H., Heinz G., Nikfardjam M., Bojic A., Schellongowski P., Angermayr B., Kitzberger R., Warszawska J. (2009). Hypoxic hepatitis: Underlying conditions and risk factors for mortality in critically ill patients. Intensive Care Med..

[29] Lee S.B., Park G.M., Lee J.Y., Lee B.U., Park J.H., Kim B.G., Jung S.W., Jeong I.D., Bang S.J., Shin J.W. (2018). Association between non-alcoholic fatty liver disease and subclinical coronary atherosclerosis: An observational cohort study. J. Hepatol..

[30] Mihm S. (2018). Danger-associated molecular patterns (damps): Molecular triggers for sterile inflammation in the liver. Int. J. Mol. Sci..

[31] Huebener P., Pradere J.P., Hernandez C., Gwak G.Y., Caviglia J.M., Mu X., Loike J.D., Jenkins R.E., Antoine D.J., Schwabe R.F. (2015). The HMGB1/rage axis triggers neutrophil-mediated injury amplification following necrosis. J. Clin. Invest..

[32] Kataoka H., Kono H., Patel Z., Kimura Y., Rock K.L. (2014). Evaluation of the contribution of multiple damps and damp receptors in cell death-induced sterile inflammatory responses. PLoS ONE.

[33] Colussi G., Zuttion F., Bais B., Dolso P., Valente M., Gigli G.L., Gasparini D., Sponza M., Catena C., Sechi L.A. (2018). Pre-procedural statin use is associated with improved long-term survival and reduced major cardiovascular events in patients undergoing carotid artery stenting: A retrospective study. J. Clin. Med..

[34] Cirillo P., Pacileo M., de Rosa S., Calabro P., Gargiulo A., Angri V., Prevete N., Fiorentino I., Ucci G., Sasso L. (2007). HMG-CoA reductase inhibitors reduce nicotine-induced expression of cellular adhesion molecules in cultured human coronary endothelial cells. J. Vasc. Res..

[35] Schmidt M., Lamberts M., Olsen A.M., Fosboll E., Niessner A., Tamargo J., Rosano G., Agewall S., Kaski J.C., Kjeldsen K. (2016). Cardiovascular safety of non-aspirin non-steroidal anti-inflammatory drugs: Review and position paper by the working group for cardiovascular pharmacotherapy of the european society of cardiology. Eur. Heart J..

[36] Mythili S., Malathi N. (2015). Diagnostic markers of acute myocardial infarction. Biomed. Rep..

[37] Bodor G.S. (2016). Biochemical markers of myocardial damage. EJIFCC.

[38] Pacileo M., Cirillo P., de Rosa S., Ucci G., Petrillo G., Musto D’Amore S., Sasso L., Maietta P., Spagnuolo R., Chiariello M. (2007). The role of neopterin in cardiovascular disease. Monaldi Arch. Chest Dis..

[39] Cirillo P., Golino P., Calabro P., Cali G., Ragni M., de Rosa S., Cimmino G., Pacileo M., de Palma R., Forte L. (2005). C-reactive protein induces tissue factor expression and promotes smooth muscle and endothelial cell proliferation. Cardiovasc. Res..

